# Computational analysis of control of hepatitis B virus disease through vaccination and treatment strategies

**DOI:** 10.1371/journal.pone.0288024

**Published:** 2023-10-26

**Authors:** Azhar Iqbal Kashif Butt, Muhammad Imran, Javeria Aslam, Saira Batool, Saira Batool

**Affiliations:** 1 Department of Mathematics and Statistics, College of Science, King Faisal University, Al-Ahsa, Saudi Arabia; 2 Department of Mathematics, GC University, Lahore, Pakistan; 3 Tandy School of Computer Science, The University of Tulsa, Tulsa, OK, United States of America; 4 Government Associate College (W) Kamar Mashani, Mianwali, Pakistan; Virginia Commonwealth University, UNITED STATES

## Abstract

Hepatitis B disease is an infection caused by a virus that severely damages the liver. The disease can be both acute and chronic. In this article, we design a new nonlinear SVEICHR model to study dynamics of Hepatitis B Virus (HBV) disease. The aim is to carry out a comprehensive mathematical and computational analysis by exploiting preventive measures of vaccination and hospitalization for disease control. Mathematical properties of proposed model such as boundedness, positivity, and existence and uniqueness of the solutions are proved. We also determine the disease free and endemic equilibrium points. To analyze dynamics of HBV disease, we compute a biologically important quantity known as the reproduction number R_0_ by using next generation method. We also investigate the stability at both of the equilibrium points. To control the spread of disease due to HBV, two feasible optimal control strategies with three different cases are presented. For this, optimal control problem is constructed and Pontryagin maximum principle is applied with a goal to put down the disease in the population. At the end, we present and discuss effective solutions obtained through a MATLAB code.

## 1. Introduction

Epidemiology is the study of health and disease as well as the causes associated with them at the population level. Hepatitis B is an epidemiological disease caused by a hepatitis B virus. It simply refers to liver inflammation [1]. Generally, most people do not experience symptoms until the infection becomes fatal. Therefore, hepatitis B virus is called the silent killer. However, the common symptoms of HBV are fever, fatigue, nausea, vomiting, belly pain, and joint pain [1–3]. The incubation period of HBV is from 1.5 to 6 months (average 4 months) [2, 3].

In 1942, a hepatitis outbreak took place that affected 28,585 soldiers. The soldiers were infected with virus after getting yellow fever vaccine [4]. During second world war, there were possibly 16 million cases of hepatitis. Every year, around 4.5 million new HBV infections arise worldwide with one-fourth of these progressing to liver damage. Mccallum and Bauer first time coined the terms Hepatitis-A and Hepatitis-B in 1947 to distinguish between infectious (epidemic) and serum hepatitis [5]. The World Health Organization (WHO) scientific group working on viral hepatitis adopted these terms in 1973. Blumberg et al. [6] discovered at the National Institutes of Health (USE) that the serum of an Australian person has a precipitating antigen on agar gel with the serum of a patient who has received multiple blood transfusions; this protein is now known as “Hepatitis-B surface antigen HBsAg,” and they named the protein “Australian antigen-Au antigen.” It became clear over time that this protein is linked to type B hepatitis. Researchers led by Prince, Okochi, and Murakam discovered Au antigen (hepatitis B surface antigen) only in the serum of patients infected with type B hepatitis in 1968 [7]. Acute hepatitis B has a fatality rate of 0.5-1%. About 6,00,000 people die every year due to HBV infection [8–11].

Possible forms of transmission of HBV include vertical transmission (mother to baby) and horizontal transmission (exposure to infected blood or any other body fluid) [12]. There are two forms of hepatitis B virus: acute and chronic. The acute hepatitis B virus does not persist more than 6 months (with or without symptoms) and the infected individual can transmit the disease to others. In this case, our immune system is capable of removing the HBV from the body. On the other hand, chronic hepatitis B virus takes 6 months or longer. In this case, our immune system is unable to eliminate the HBV. The main difference between acute and chronic/carriers HBV infection is the duration of the infection. Acute HBV infection is a short-term illness that typically resolves on its own, while chronic HBV infection lasts longer than six months and can cause serious liver damage over time. There are several treatments but not a specific treatment for acute infectious hepatitis B virus. Therefore, care is aimed at maintaining comfort and adequate nutritional balance, including replacement of fluids lost from vomiting and diarrhoea. The other treatments for acute hepatitis B virus are, Hepatitis B immunoglobulin (HBIG) and in some cases, HBIG is also helpful to prevent from becoming this acute to chronic/carrier HBV infection. Chronic hepatitis B infection can be treated with medications, including antiviral agents taken orally. Cirrhosis treatment can slow the progression of the disease, lower the incidence of liver cancer, and improve long-term survival [13]. Depending on the setting and eligibility criteria, WHO estimates that 12% to 25% of people with chronic hepatitis B infection took treatment in 2021 [14]. The World Health Organization recommends oral treatments (tenofovir or entecavir) as the most effective drugs for hepatitis B virus suppression. Most people who begin hepatitis B treatment must continue it for the rest of their lives. Other treatments for chronic or carriers include antiviral medication, interferon therapy, and, in the worst-case scenario, liver transplantation. We should have proper treatment otherwise it can lead to major health problems and possibly be fatal [15–17].

The mathematical modeling is a useful means that relates real-world problems to mathematical equations. A model gives us a comprehensive picture of the possible outcomes of the disease [18–21]. Recognizing the severity of HBV disease, many researchers and epidemiological scientists of the world started working in understanding and analyzing the disease dynamics and control patterns. A variety of epidemiological models exist in the literature [22–29] to probe and to control the disease in the human population. The ultimate aim in these articles was to study the disease spread patterns, virus transmission modes, damages caused by virus and the possible control or prevention strategies.

Since HBV is one of the major global health problems and the viral disease is affecting millions of people every year, it is necessary to explore the disease for best possible treatments. Controlling HBV disease is still a concern of the world as many governments especially the third world countries are trying to eradicate the disease from the society. The purpose of this manuscript is to design some realistic optimal control strategies and to study the impact of these strategies in controlling the spread of disease. For this purpose, we design a new *SVEICHR* model for HBV disease where the addition of vaccination compartment *V* and the hospitalization compartment *H* is considered for the sake of disease control and treatment. A similar strategy for disease control is used in [16, 17]. However, in [16] the considered model does not include the vaccination and hospitalization compartments whereas in [17] the model is considered with vaccination and treatment compartments but with different disease flow patterns and controls.

In [17], the same compartmental control strategies were applied, as we have previously mentioned. However, there are several gaps in the study which we aim to address in our proposed model. Specifically, the authors in [17] did not account for the interaction between vaccinated and infectious individuals, particularly those with chronic conditions, which is not a realistic approach. In addition, the absence of vertical remission from mother to child is another unrealistic assumption. Although Alrabaiah et al. in [17] employed treatment for acute and chronic cases in their control strategies, but in model they considered the recovery rate of under-treated individuals as a treatment strategy. Before applying numerical techniques, the considered model was not verified for the essential well-posed property, as the existence and uniqueness of the solution was not demonstrated in [17]. To address these issues, we propose a mathematical model with more realistic assumptions. In our proposed model, direct interaction between vaccinated and infectious individuals is possible, which can result in virus transmission to low-immunity vaccinated individuals. Therefore, even vaccinated people can be exposed to the virus. Moreover, we consider the transmission of the virus through vertical transmission from chronically infected mothers to their newborn babies. We will investigate the validity of our proposed model by demonstrating its characteristic properties, including positivity and boundedness of the solutions. In order to establish the well-posedness of the suggested model, we will demonstrate the existence of a unique solution and conduct stability analysis of the disease-free and endemic equilibrium points. Additionally, we design an optimal control problem to address the control of HBV disease and to determine the optimal vaccination rate for susceptible individuals as well as the optimal treatment rates for those with acute and chronic/carriers hepatitis B virus. To further elucidate the attributes of the control problem, we perform various numerical simulations using an optimization algorithm.

In Section 2, we develop an *SVEICHR* model for HBV disease control. Section 3 deals with the important property for a system of ordinary differential equations, the existence and uniqueness of a solution as well as the fundamental properties of the HBV model i.e., boundedness and positivity of the solutions. We find out the model’s disease free and endemic equilibrium points and to check the transmission dynamics of HBV disease, we compute the biologically important property i.e., the reproduction number R_0_ [30–33] in section 4. In Section 5, we establish the local and global stabilities of the developed model at both of the equilibrium points. In Section 6, we perform an optimal control analysis to control the dynamical behavior of HBV disease at the minimum cost of implemented strategies [16, 17, 34, 35]. For this, we define an objective functional involving state and control variables and implement the Pontryagin Maximum Principle [36] to optimize the control problem. We employ a well known RK-4 method to obtain optimal numerical solutions that validate the analytical results of the proposed model. Graphical simulations along with detailed discussions are also part of Section 6. Our study is summarized in section 7.

## 2. Model formulation

Hepatitis B is a virus-borne infection caused by HBV which assaults and damages the liver. To develop a mathematical model for HBV disease, we divide the total population into seven compartments. *N*(*t*) is used to represent the total population at any time *t* that is sort out into seven times-dependent classes. The first class is susceptible class *S*(*t*), the class which is healthy but can be effected by the disease. The second class is vaccinated class *V*(*t*). The third class is exposed class *E*(*t*), the class which is infected but didn’t transfer the disease. The fourth class is acute infectious individuals *I*(*t*), the class which contracts with the disease and then stick with it. The fifth class is chronic HBV carriers *C*(*t*), the class which is the cause of transmission of chronic HBV in the population. The sixth class is hospitalized class *H*(*t*), the class which contains individual with severe conditions. At the end, seventh class is recovered *R*(*t*), the class which has immunity against the disease either through vaccination or with the human body’s immune system. Thus, the total population at any time *t* is given as

$$\begin{array}{c} N(t)=S(t)+V(t)+E(t)+I(t)+C(t)+H(t)+R(t). \end{array}$$ (1)

**Fig 1:**
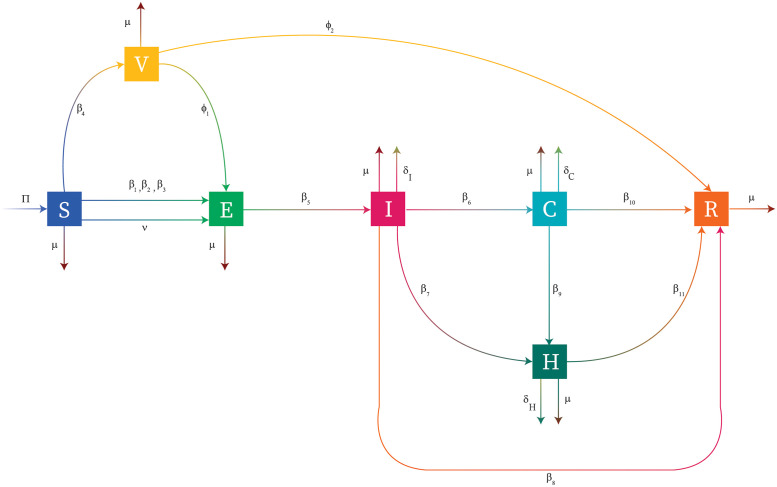
Flow diagram. HBV disease transmission through compartments.

Flow pattern of the disease as well as the connection between the compartments *S*, *V*, *E*, *I*, *C*, *H*, *R* along with transmission rates is shown in the Fig 1.

The system of nonlinear ODEs describing the disease flow pattern of Fig 1 can be written in the form:

$$\begin{array}{cc} \frac{dS}{dt} & =\Pi -\left(\beta_{1}I+\left(\beta_{2}+\nu \right)C+\beta_{3}H\right)S-\beta_{4}S-\mu S,\\ \frac{dV}{dt} & =\beta_{4}S-\phi_{1}VI-\left(\phi_{2}+\mu \right)V,\\ \frac{dE}{dt} & =(\beta_{1}I+\left(\beta_{2}+\nu \right)C+\beta_{3}H)S+\phi_{1}VI-\left(\beta_{5}+\mu \right)E,\\ \frac{dI}{dt} & =\beta_{5}E-\left(\beta_{6}+\beta_{7}+\beta_{8}+\mu +\delta_{I}\right)I,\\ \frac{dC}{dt} & =\beta_{6}I-\left(\beta_{9}+\beta_{10}+\mu +\delta_{C}\right)C,\\ \frac{dH}{dt} & =\beta_{7}I+\beta_{9}C-\left(\beta_{11}+\delta_{H}+\mu \right)H,\\ \frac{dR}{dt} & =\beta_{8}I+\beta_{10}C+\beta_{11}H+\phi_{2}V-\mu R, \end{array}$$ (2)

along with the set of non-negative initial conditions:

$$\begin{array}{c} S\left(0\right)=S_{0},\;V\left(0\right)=V_{0},\;E\left(0\right)=E_{0},\;I\left(0\right)=I_{0},\;C\left(0\right)=C_{0},\;H\left(0\right)=H_{0},\;R\left(0\right)=R_{0}. \end{array}$$

where *S*_0_, *V*_0_, *E*_0_, *I*_0_, *C*_0_, *H*_0_, *R*_0_ are non-negative initial populations for the respective compartments. Detailed description of the parameters involved in model (2) and their values are given in the Table 1.

**Table 1:** Parameters description with their numerical values.

Parameter	Description	Value	Source
*Π*	Rate of new arrivals	0.0121	[5, 13, 16]
*μ*	Rate of death naturally	0.0121	[16]
*β*_1_	Rate of switching from class *S* to *E* after contact with *I*	0.65	Assumed
*β*_2_	Rate of shifting from class *S* to *E* after contact with *C*	0.5	[16]
*β*_3_	Rate of switching from class *S* to *E* after contact with *H*	0.36	[16]
*β*_4_	Rate of vaccinated susceptible	0.2	[22]
*β*_5_	Rate of shifting from class *E* to *I*	0.1989	[5]
*β*_6_	Rate of shifting from class *I* to *C*	0.025	[16]
*β*_7_	Rate of shifting from class *I* to *H*	0.2	Estimated
*β*_8_	Rate of shifting from class *I* to *R*	0.36	Estimated
*β*_9_	Rate of shifting from class *C* to *H*	0.5	Assumed
*β*_10_	Rate of switching from *C* to *R*	0.07	[5]
*β*_11_	Rate os switching from *H* to *R*	0.1	Assumed
*ϕ*_1_	Rate of transmission from *V* to *E* after contact with *I*	0.06	Assumed
*ϕ*_2_	Rate of transmission from *V* to *R*	0.7	[9]
*ν*	Vertical transmission of virus from mother to child	0.035	Assumed
*δ*_*I*_	Fatality rate in *I* due to disease	0.54	Estimated
*δ*_*C*_	Fatality rate in *C* due to disease	0.05	Estimated
*δ*_*H*_	Fatality rate in *H* due to disease	0.023	Assumed

## 3. Existence and uniqueness of the solution

In this section, we state some fundamental theorems to prove that the HBV model (2) has a unique solution. Some basic definitions from functional analysis are also presented here to support the proof of stated theorems. Since the proposed HBV model (2) is autonomous, it can be written as:

$$\begin{array}{c} \frac{dZ}{dt}=T\left(Z\left(t\right)\right),\;\;\;Z\left(0\right)=Z_{0}, \end{array}$$ (3)

where *Z*(*t*) ∈ *C*^1^[0, *T*_*f*_] and $Z\left(t\right):R_{+}\to R_{+}^{7}$ is a real valued function defined by

$$Z\left(t\right)={\left(S(t),V(t),E(t),I(t),C(t),H(t),R(t)\right)}^{T},$$

along with

$$Z_{0}={\left(S(0),V(0),E(0),I(0),C(0),H(0),R(0)\right)}^{T},$$

and

$$T\left(t\right)={\left(T_{1}\left(Z\right),T_{2}\left(Z\right),T_{3}\left(Z\right),T_{4}\left(Z\right),T_{5}\left(Z\right),T_{6}\left(Z\right),T_{7}\left(Z\right)\right)}^{T},$$

where T(t) is a column vector and $T_{i}\left(Z\right),\;i=1,2,\ldots ,7$ are its components, $T_{i}\left(Z\right),\;i=1,2,\ldots ,7$ represent the right hand sides of the equations of model (2). To establish the existence and uniqueness for the solution of model (3), we state some basic theorems and definitions.

**Theorem 1** [37] *Let* *h* : *D* → *R*^*n*^ *be a continuously differentiable mapping from* *D* ⊆ *R* *to* *R*^*n*^, *x* ∈ *D*. *Then* *h* *satisfies a Lipschitz condition on each convex compact subset* D *of* *D* *with Lipchitz constant* *K*. *Where* *K* *is the supremum of the derivative of h on* D, *i.e.*,

$$K=\underset{x\in D}{\text{sup}}\;|\frac{dh}{dx}|.$$

**Theorem 2** [38] *Suppose* *D* = {(*t*, *z*)|*t* ∈ *R*, *z* ∈ *R*^*n*^}, *and let* *h*(*t*, *z*) *be continuous on* *D* *and satisfies Lipschitz condition there, then the initial value problem*

$$\begin{array}{c} \frac{dz}{dt}=h\left(t,z\right),\;z\left(t_{0}\right)=z_{0}, \end{array}$$

*has a solution*.

**Definition 1** [39] *A sequence* (*x*_*n*_) *in a metric space* *X* = (*X*, *d*) *is said to be Cauchy if for every* *ϵ* > 0 *there is an* *N* = *N*(*ϵ*) *such that*

$$d\left(x_{n},x_{m}\right)<\epsilon ,\;for\;every\;m,n>n_{0}\in N.$$

**Definition 2** [39] *A sequence* (*x*_*n*_) *is contractive in a metric space X if there exist a constant* *C* ∈ (0, 1) *such that*

$$d\left(x_{n},x_{n-1}\right)<Cd(x_{n-1},x_{n-2}),$$

*for all* *n* ∈ *N* *and* *C* *is called contractive constant of the sequence*.

**Theorem 3** [39] *A sequence is convergent in a complete metric space X iff it is Cauchy*.

**Theorem 4** [39] *Every contractive sequence is a Cauchy sequence, and therefore convergent in complete metric space*.

**Theorem 5** *The function* T(Z) in (3) *is Lipschitz continuous*.

**Proof**: Let V be a convex compact subset of

$$D=\{\left(Z\left(t\right)\right)|\;t_{0}\leq t\leq T_{f},\;Z\in R_{+}^{7}\}.$$

Let $Z_{1},Z_{2}\in V$, then by Mean Value Theorem ∃ *ζ* ∈ (*Z*_1_, *Z*_2_) such that

$$\begin{array}{c} \frac{T\left(Z_{1}\left(t\right)\right)-T\left(Z_{2}\left(t\right)\right)}{Z_{1}\left(t\right)-Z_{2}\left(t\right)}=g^{{}^{\prime }}\left(\zeta \left(t\right)\right), \end{array}$$

or

$$\begin{array}{c} T\left(Z_{1}\left(t\right)\right)-T\left(Z_{2}\left(t\right)\right)=T^{{}^{\prime }}\left(\zeta \left(t\right)\right).\left(Z_{1}\left(t\right)-Z_{2}\left(t\right)\right), \end{array}$$

and hence,

$$\begin{array}{cc} |T\left(Z_{1}\left(t\right)\right)-T\left(Z_{2}\left(t\right)\right)| & =|T^{{}^{\prime }}\left(\zeta \left(t\right)\right).Z_{1}\left(t\right)-Z_{2}\left(t\right)|,\\ & \leq \|T^{{}^{\prime }}{\left(\zeta \right)\|}_{\infty }{\left\|Z_{1}-Z_{2}\right\|}_{\infty }. \end{array}$$

Since $T\in C^{1}\left[0,T_{f}\right]$, hence over convex compact set V, ∃ a constant λ > 0 such that

$$\begin{array}{c} \|T^{{}^{\prime }}{\left(\zeta \right)\|}_{\infty }\leq \lambda . \end{array}$$

Hence,

$$\begin{array}{c} |T\left(Z_{1}\left(t\right)\right)-T\left(Z_{2}\left(t\right)\right)|\leq \lambda \|Z_{1}-Z_{2}{\|}_{\infty },\\ \underset{t\in [0,T_{f}]}{\text{sup}}\;|T\left(Z_{1}\left(t\right)\right)-T\left(Z_{2}\left(t\right)\right)|\leq \lambda \|Z_{1}-Z_{2}{\|}_{\infty }, \end{array}$$

or it can be written as

$$\begin{array}{c} \|T\left(Z_{1}\right)-T\left(Z_{2}\right){\|}_{\infty }\leq \lambda \|Z_{1}-Z_{2}{\|}_{\infty }. \end{array}$$

Hence T(Z) is Lipschitz.

**Theorem 6** *Suppose that the function* T(Z) *satisfies the Lipschitz condition*

$$\begin{array}{c} \|T\left(Z_{2}\right)-T\left(Z_{1}\right){\|}_{\infty }\leq \lambda {\left\|Z_{2}-Z_{1}\right\|}_{\infty }, \end{array}$$

*then the problem* (3) *has a unique solution for*

$$\begin{array}{c} k=\lambda T_{f}<1. \end{array}$$

**Proof.** By fundamental theorem of calculus, solution of IVP (3) can be written in the form:

$$\begin{array}{c} Z\left(t\right)=Z\left(0\right)+\int_{0}^{T_{f}}T\left(Z\right)dt. \end{array}$$ (4)

We will prove that the function *u*(*t*) is a solution of (3) iff it satisfies the integral Eq (4). Let *u*(*t*) be the solution of (3), then by fundamental theorem of calculus we can write

$$\begin{array}{c} u\left(t\right)=u\left(0\right)+\int_{0}^{T_{f}}T\left(u\right)dt. \end{array}$$ (5)

That is equivalent to (4). For converse implication, we let *u*_*n*_(*t*) is a sequence of solutions which converges to the solution (3) with successive iterative form and defined as:

$$\begin{array}{c} u_{n}\left(t\right)=u_{0}\left(t\right)+\int_{0}^{T_{f}}T\left(u_{n-1}\left(t\right)\right)dt,\;\;i=1,2,.,.,n, \end{array}$$ (6)

with *u*_0_(*t*) = *u*(0). First of all we show that the sequence (6) is contractive if *k* = *λT*_*f*_ < 1. Consider

$$\begin{array}{cc} |u_{n}\left(t\right)-u_{n-1}\left(t\right)|= & |\int_{0}^{T_{f}}\left[T\left(u_{n-1}\left(t\right)\right)-T\left(u_{n-2}\left(t\right)\right)\right]dt|,\\ & \leq \int_{0}^{T_{f}}|T\left(u_{n-1}\left(t\right)\right)-T\left(u_{n-2}\left(t\right)\right)|dt,\\ & \leq \int_{0}^{T_{f}}\lambda |u_{n-1}\left(t\right)-u_{n-2}\left(t\right)|dt. \end{array}$$

Using the Lipchitzian property of the function T, we have

$$\begin{array}{cc} |u_{n}\left(t\right)-u_{n-1}\left(t\right)| & \leq \int_{0}^{T_{f}}\lambda \underset{t\in [0,T_{f}]}{\text{sup}}\;|u_{n-1}\left(t\right)-u_{n-2}\left(t\right)|dt,\\ & \leq \lambda \underset{t\in [0,T_{f}]}{\text{sup}}\;|u_{n-1}\left(t\right)-u_{n-2}\left(t\right)|\int_{0}^{T_{f}}dt,\\ & \leq \;\lambda T_{f}\underset{t\in [0,T_{f}]}{\text{sup}}\;|u_{n-1}\left(t\right)-u_{n-2}\left(t\right)|,\\ \|u_{n}-u_{n-1}{\|}_{\infty } & \leq \lambda T_{f}{\left\|u_{n-1}-u_{n-2}\right\|}_{\infty },\\ \|u_{n}-u_{n-1}{\|}_{\infty } & \leq k\|u_{n-1}-u_{n-2}{\|}_{\infty }, \end{array}$$

this implies

$$\begin{array}{c} d\left(u_{n},u_{n-1}\right)\leq k\;d\left(u_{n-1},u_{n-2}\right). \end{array}$$ (7)

Thus, Eq (7) implies that the sequence (6) is contractive, hence Theorem 4 verifies it as a Cauchy sequence. Now for *m*, *n* ∈ *N* and *m* > *n*,

$$\begin{array}{cc} \|u_{m}-u_{n}\|= & \|u_{m}-u_{m-1}+u_{m-1}-u_{m-2}+u_{m-2}...-u_{n+1}+u_{n+1}-u_{n}+u_{n}-u_{n}\|,\\ & \leq \|u_{m}-u_{m-1}\left\|+\right\|u_{m}-1-u_{m-2}\left\|+...+\|u_{n+1}-u_{n}\right\|,\\ & \leq k^{m-1}\|u_{1}-u_{0}\|+k^{m-2}\|u_{1}-u_{0}\left\|+...+k^{n}\|u_{1}-u_{0}\right\|,\\ & \leq \left[k^{m-1}+k^{m-2}+...+k^{n}\right]\|u_{1}-u_{0}\|, \end{array}$$

where

$$k=\lambda T_{f}<1.$$

Hence, right hand side is geometric series which is always convergent for |*k*|<1.

$$\begin{array}{c} \|u_{m}-u_{n}\|\leq k^{n}\frac{1-k^{m-n}}{1-k}\|u_{1}-u_{0}\left\|\leq k^{n}\frac{1}{1-k}\|u_{1}-u_{0}\right\|. \end{array}$$

Since 0 < *k* < 1, lim_*n*→∞_(*k*^*n*^) = 0. Therefore we infer that sequence (*u*_*n*_) is Cauchy and hence from Theorem 3 it convergent. Let lim_*n*→∞_(*u*_*n*_) = *u*, then Eq (6) gives

$$\begin{array}{c} lim_{n\to \infty }u_{n}\left(t\right)=u\left(t\right)=u\left(0\right)+\int_{0}^{T_{f}}T\left(u\left(t\right)\right)dt. \end{array}$$ (8)

Eq (8) is the required solution.

### Uniqueness

To prove uniqueness of the solution we suppose on contrary that the sequence (*u*_*n*_) converges to two different limits *u*_1_ and *u*_2_. Then there exist *n*_1_*and* *n*_2_ ∈ *N* such that,

$$\begin{array}{c} \|u_{n}-u_{1}\|<\epsilon_{1},\;n_{1}\geq n,\\ \|u_{n}-u_{2}\|<\epsilon_{2},\;n_{2}\geq n. \end{array}$$

Let n* = *max*{*n*_1_, *n*_2_}, then

$$\begin{array}{c} \|u_{1}-u_{2}\left\|=\right\|u_{1}-u_{n}+u_{n}-u_{2}\left\|\leq \right\|u_{1}-u_{n}\left\|+\|u_{n}-u_{2}\right\|<\epsilon_{1}+\epsilon_{2}=\epsilon , \end{array}$$

which implies,

$$\begin{array}{c} \|u_{1}-u_{2}\|=0. \end{array}$$

Hence, we have proved that solution (8) of IVP (3) exists and is unique.

### 3.1 Boundedness and positivity

To examine the fundamental properties of Hepatitis B virus model, we show that in a feasible region, for all *t* ≥ 0, the state variables *Z* = (*S*, *V*, *E*, *I*, *C*, *H*, *R*) are bounded and non-negative.

**Theorem 7** *The state variables Z(t)* = (*S*, *V*, *E*, *I*, *C*, *H*, *R*) *of the model* (2) *are bounded* ∀ *t* ≥ 0.

**Proof:** By using the Eq (1), we can write

$$\frac{dN}{dt}=\frac{dS}{dt}+\frac{dV}{dt}+\frac{dE}{dt}+\frac{dI}{dt}+\frac{dC}{dt}+\frac{dH}{dt}+\frac{dR}{dt}.$$

Substituting the right hand side of the model (2) in Eq (1) and by simplification, we get the following equation that yields the rate at which the total population changes, i.e.,

$$\begin{array}{c} \frac{dN}{dt}=\Pi -\mu N-\left(\delta_{I}I+\delta_{C}C+\delta_{H}H\right). \end{array}$$ (9)

From Eq (9), we get the inequality

$$\begin{array}{c} \frac{dN}{dt}\leq \Pi -\mu N, \end{array}$$ (10)

with initial condition

$$N\left(0\right)\leq \frac{\Pi }{\mu }.$$

Applying the Laplace transformation on the Eq (10), we can write

$$L[\frac{dN(t)}{dt}]\leq L\left[\Pi \right]-\mu L[\mu N(t)].$$

$$\begin{array}{cc} s(N(s))-N(0) & \leq \frac{\Pi }{s}-\mu N\left(s\right),\\ s(N(s))+\mu N(s) & \leq \frac{\Pi }{s}+N\left(0\right),\\ N(s) & \leq \frac{\Pi }{s(s+\mu )}+\frac{N(0)}{s+\mu }. \end{array}$$

Using partial fraction technique, we get:

$$\begin{array}{c} N(s)\leq \frac{\Pi }{\mu }\frac{1}{s}-\frac{\Pi }{\mu }\frac{1}{\left(s+\mu \right)}+\frac{N(0)}{s+\mu }. \end{array}$$ (11)

Applying inverse Laplace transformation on both side of (11), we get

$$\begin{array}{c} L^{-1}(N(s))\leq \frac{\Pi }{\mu }L^{-1}(\frac{1}{s})-\frac{\Pi }{\mu }L^{-1}(\frac{1}{\left(s+\mu \right)})+N\left(0\right)L^{-1}(\frac{1}{s+\mu }), \end{array}$$

or

$$\begin{array}{cc} N(t) & \leq \frac{\Pi }{\mu }-\frac{\Pi }{\mu }\;\text{exp}\;\left(-\mu t\right)+N\left(0\right)\;\text{exp}\;\left(-\mu t\right),\\ & \leq \frac{\Pi }{\mu }-[\frac{\Pi }{\mu }-N\left(0\right)]\;\text{exp}\;\left(-\mu t\right). \end{array}$$

Hence, we can state

$$\underset{t\to \infty }{\text{lim}}\;N\left(t\right)\leq \frac{\Pi }{\mu }.$$

Thus, we proved that the state variables (*S*, *V*, *E*, *I*, *C*, *H*, *R*) of the model (2) remains bounded in the feasible region.

**Theorem 8** *Consider the model* (2) *with the given non-negative initial conditions. Then the solution* *Z*(*t*) = (*S*, *V*, *E*, *I*, *C*, *H*, *R*) *of this model* (2) *is either positive or zero* ∀*t* ≥ 0.

**Proof:** Let us consider the first equation of the model (2), i.e.,

$$\begin{array}{c} \frac{dS}{dt}=\Pi -\left(\beta_{1}I+\left(\beta_{2}+\nu \right)C+\beta_{3}H\right)S+\left(\beta_{4}+\mu \right)S. \end{array}$$ (12)

Since we proved that all the state variables are bounded, hence ∃ a finite *Υ* > 0 such that:

$$ϒ=\;\text{sup}\;[\left(\beta_{1}I+\left(\beta_{2}+\nu \right)C+\beta_{3}H\right)+\left(\beta_{4}+\mu \right)].$$

Thus,

$$\begin{array}{c} \frac{dS}{dt}\geq \Pi -ϒS(t). \end{array}$$ (13)

Applying Laplace transformation, we get:

$$\begin{array}{cc} L[\frac{dS}{dt}] & \geq L[\Pi ]-L[ϒS],\\ s(S(s))-S(0) & \geq \frac{\Pi }{s}-ϒS\left(s\right),\\ s(S(s))+ϒS(s) & \geq \frac{\Pi }{s}+S\left(0\right),\\ S(s) & \geq \frac{\Pi }{s(s+ϒ)}+\frac{S(0)}{s+ϒ}. \end{array}$$

Further simplification yields us:

$$\begin{array}{c} S(s)\geq \frac{\Pi }{ϒ}\frac{1}{s}-\frac{\Pi }{ϒ}\frac{1}{\left(s+ϒ\right)}+\frac{N(0)}{s+ϒ}. \end{array}$$ (14)

Applying inverse Laplace transformation on both side of (14), we obtain:

$$\begin{array}{cc} L^{-1}(S(s)) & \geq \frac{\Pi }{ϒ}L^{-1}(\frac{1}{s})-\frac{\Pi }{ϒ}L^{-1}(\frac{1}{\left(s+ϒ\right)})+S\left(0\right)L^{-1}(\frac{1}{s+ϒ}),\\ S(t) & \geq \frac{\Pi }{ϒ}-\frac{\Pi }{ϒ}\;\text{exp}\;\left(-ϒt\right)+S\left(0\right)\;\text{exp}\;\left(-ϒt\right). \end{array}$$ (15)

Since 0 ≤ exp(−*Υt*) ≤ 1, hence $\frac{\Pi }{ϒ}\geq \frac{\Pi }{ϒ}\;\text{exp}\;\left(-ϒt\right)$ and also *S*(0)exp(−*Υt*) ≥ 0.

Thus, it is obvious from the Eq (15), *S*(*t*) ≥ 0, for all *t* ≥ 0. By using similar approach, we can easily show that all other state variables *Z*(*t*) ≥ 0, ∀*t* ≥ 0.

## 4. Equilibrium points

Equilibrium points are computed by solving the the steady-state equations of the model (2) by considering the absence and presence of hepatitis B virus in the community.

Therefor, HBV free or the disease free equilibrium (DFE) point is computed to give:

$$\begin{array}{cc} P_{0}= & (S^{0},V^{0},E^{0},I^{0},C^{0},H^{0},R^{0}),\\ = & (\frac{\Pi }{\mu +\beta_{4}},\frac{\beta_{4}\Pi }{\left(\phi_{2}+\mu \right)\left(\mu +\beta_{4}\right)},0,0,0,0,\frac{\phi_{1}\beta_{4}\Pi }{\left(\mu \right)\left(\phi_{2}+\mu \right)\left(\mu +\beta_{4}\right)}), \end{array}$$

and HBV present or the endemic equilibrium (EE) point is given as:

$$\begin{array}{c} P_{1}=(S^{1},V^{1},E^{1},I^{1},C^{1},H^{1},R^{1}), \end{array}$$

where

$$\begin{array}{cc} S^{1}= & \frac{\Pi }{\alpha }>0,\;E^{1}=\frac{1}{\beta_{5}}(j_{4}I^{1})>0,\\ V^{1}= & \frac{j_{4}}{\beta_{5}}-[(\beta_{1}+\frac{(\beta_{2}+\nu )\beta_{6}}{j_{5}})+\frac{\beta_{3}}{j_{6}}(\beta_{7}+\frac{\beta_{6}\beta_{9}}{j_{5}})]S^{1}>0,\\ I^{1}= & \frac{1}{\phi_{1}V^{1}}[\beta_{4}S^{1}-(\phi_{2}+\mu )V^{1}]>0,\;C^{1}=\frac{\beta_{6}I^{1}}{j_{5}}>0,\;H^{1}=\frac{\beta_{7}+\frac{\beta_{6}\beta_{9}}{j_{5}}}{j_{6}}I^{1}>0,\\ R^{1}= & \frac{\beta_{8}I^{1}+\beta_{10}C^{1}+\beta_{11}H^{1}+\phi_{2}V^{1}}{\mu }>0, \end{array}$$

along with

$$\begin{array}{cc} \alpha & =\beta_{1}I^{1}+\left(\beta_{2}+\nu \right)C^{1}+\beta_{3}H^{1}+\left(\beta_{4}+\mu \right),\;j_{1}=\beta_{4}+\mu ,\;j_{2}=\phi_{2}+\mu ,\;j_{3}=\beta_{5}+\mu ,\\ j_{4} & =\beta_{6}+\beta_{7}+\beta_{8}+\mu +\delta_{I},\;j_{5}=\beta_{9}+\beta_{10}+\delta_{C}+\mu ,\;j_{6}=\beta_{11}+\delta_{H}+\mu . \end{array}$$

### 4.1 Reproduction number

The reproduction number R_0_ is the estimated rate of a disease’s transmission capability and for disease free equilibrium stability it gives a mathematical criterion. It represents the average number or mean of new infections that are produced by an infected case throughout the population. It also generates the criteria for the stability of the system. The next-generation matrix technique which is introduced by Diekmann and Heesterbeek in 1990 [33, 34], is used to formulate and compute reproduction number R_0_. Basically R_0_ is a spectral radius of the matrix $F{\bar{V}}^{-1}$, where *F* represents the Jacobian of the rate of new arrivals in the infected classes and the Jacobian of terms that are remaining in the infectious compartments is denoted by $\bar{V}$.

To compute R_0_, at first, we decompose the right-hand sides of the infection carrying differential equations of the mathematical model (2) as F-V. where F represents the rate of new arrivals(transmission terms) in the infected classes and the remaining (translation terms) in the infectious compartments is denoted by V. So, for the computation of R_0_, we consider the infection carrying equations of the mathematical model (2) and write the corresponding column matrix F of arrival rates for new infected individuals, i.e.,

$$F=(\begin{array}{c} -\phi_{1}VI\\ (\beta_{1}I+\left(\beta_{2}+\nu \right)C+\beta_{3}H)S+\phi_{1}VI\\ 0\\ 0\\ 0 \end{array}),$$

and the transitional terms of the considered equations are represented by the column matrix V as

$$V=(\begin{array}{c} -\beta_{4}S+\left(\phi_{2}+\mu \right)V\\ (\beta_{5}+\mu )E\\ -\beta_{5}E+\left(\beta_{6}+\beta_{7}+\beta_{8}+\mu +\delta_{I}\right)I\\ -\beta_{6}I+\left(\beta_{9}+\beta_{10}+\mu +\delta_{C}\right)C\\ -\beta_{7}I-\beta_{9}C+\left(\beta_{11}+\delta_{H}+\mu \right)H \end{array}).$$

The Jacobian matrix *F* is obtained by taking the derivatives of F at DFE point *P*_0_ with respect to state variables *Z*_*i*_ = (*V*, *E*, *I*, *C*, *H*), i.e.,

$$F={\left(\frac{\partial F_{i}}{\partial z_{i}}\right)}_{P_{0}},\;i=1,2,3,4,5,$$

and the Jacobian matrix *F* is written as:

$$F={\left(\begin{array}{ccccc} 0 & 0 & -\phi_{1}V^{0} & 0 & 0\\ 0 & 0 & \phi_{1}V^{0}+\beta_{1}S^{0} & \left(\beta_{2}+\nu \right)S^{0} & \beta_{3}S^{0}\\ 0 & 0 & 0 & 0 & 0\\ 0 & 0 & 0 & 0 & 0\\ 0 & 0 & 0 & 0 & 0 \end{array}\right)}_{p_{0}}.$$

Similarly the derivatives of V at DFE point *P*_0_ presented by

$$\bar{V}={\left(\frac{\partial V_{i}}{\partial z_{i}}\right)}_{P_{0}},$$

yields the below Jacobian matrix $\bar{V}$,

$$\bar{V}={\left(\begin{array}{ccccc} j_{2} & 0 & 0 & 0 & 0\\ 0 & j_{3} & 0 & 0 & 0\\ 0 & -\beta_{5} & j_{4} & 0 & 0\\ 0 & 0 & -\beta_{6} & j_{5} & 0\\ 0 & 0 & -\beta_{7} & -\beta_{9} & j_{6} \end{array}\right)}_{p_{0}},$$

where (*z*_1_, *z*_2_, *z*_3_, *z*_4_) = (*V*, *E*, *I*, *C*, *H*).

The absolute maximum eigenvalue of the matrix $F{\bar{V}}^{-1}$ is computed to give the reproduction number:

$$\begin{array}{c} R_{0}=\frac{\beta_{5}[j_{5}j_{6}V^{0}\phi_{1}+j_{5}j_{6}S^{0}\beta_{1}+S^{0}\left(\beta_{2}+\nu \right)\beta_{6}j_{6}+S^{0}\beta_{3}\beta_{7}j_{5}+S^{0}\beta_{3}\beta_{6}\beta_{9}]}{j_{3}j_{4}j_{5}j_{6}}. \end{array}$$ (16)

**Fig 2:**
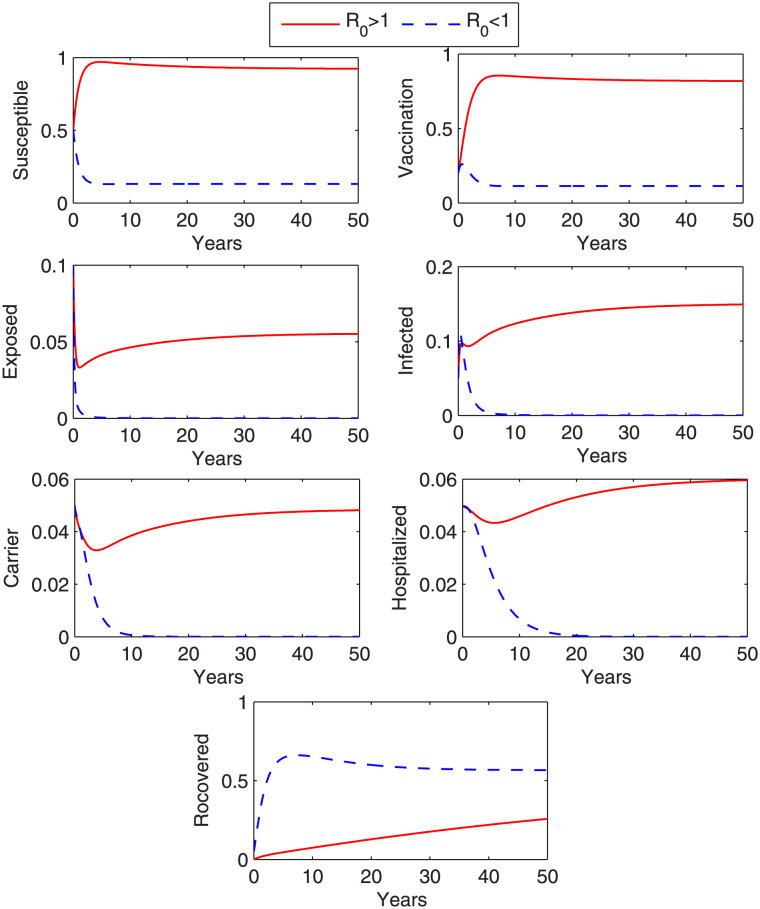
Local stability at DEF. Behavior of state variables for R_0_ < 1 and for R_0_ > 1.

The reproduction number is the number of the secondary cases generated in the population by one infected individual. The transmission potential of a disease is based on the reproduction number. If R_0_ < 1, then the transmission rate of disease is very slow in the population. In this case, the disease will gradually deteriorate and eventually vanish. If R_0_ = 1, then there is only one secondary case produced by an infected individual, therefore disease remains constant in the population through out the disease period. If R_0_ > 1, then disease spread in population more rapidly [33, 40]. Behavior of the state variables for R_0_ < 1 and R_0_ > 1 is shown in Fig 2. To compute the solution curves, we used RK-4 method along with the values of the physical parameters given in Table 1.

## 5. Stability analysis

This section deals with the local and global stabilities of the HBV model (2) at the DFE and EE points. Global stabilities are investigated using the Lyapunov theory with LaSalle invariant principle [18, 25, 26] and Castillo-Chavez approach [41].

### 5.1 Local stability at DFE

**Theorem 9** *Model* (2) *is locally asymptotically stable at* *P*_0_ *if* R_0_ < 1 *and unstable if* R_0_ > 1.

**Proof:** Corresponding to the model (2), the Jacobian matrix at disease free equilibrium point *P*_0_ is stated as:

$$J\left(P_{0}\right)=(\begin{array}{ccccccc} -j_{1} & 0 & 0 & -\beta_{1}S^{0} & -\left(\beta_{2}+\nu \right)S^{0} & -\beta_{3}S^{0} & 0\\ \beta_{4} & -j_{2} & 0 & -\phi_{1}V^{0} & 0 & 0 & 0\\ 0 & 0 & -j_{3} & \beta_{1}S^{0}+\phi_{1}V^{0} & \left(\beta_{2}+\nu \right)S^{0} & \beta_{3}S^{0} & 0\\ 0 & 0 & \beta_{5} & -j_{4} & 0 & 0 & 0\\ 0 & 0 & 0 & \beta_{6} & -j_{5} & 0 & 0\\ 0 & 0 & 0 & \beta_{7} & \beta_{9} & -j_{6} & 0\\ 0 & \phi_{2} & 0 & \beta_{8} & \beta_{10} & \beta_{11} & -\mu \end{array}).$$

We compute the following eigenvalues of Jacobian matrix *J*(*P*_0_),

$$\begin{array}{c} \lambda_{1}=-j_{1}, \end{array}$$ (17a)

$$\begin{array}{c} \lambda_{2}=-j_{2}, \end{array}$$ (17b)

$$\begin{array}{c} \lambda_{3}=-j_{3}, \end{array}$$ (17c)

$$\begin{array}{c} \lambda_{4}=\frac{-j_{4}j_{3}+\beta_{5}\phi_{1}V^{0}+\beta_{5}S^{0}\beta_{1}}{j_{3}}, \end{array}$$ (17d)

$$\begin{array}{c} \lambda_{5}=\frac{(1-R_{0})+M}{\lambda_{4}j_{6}j_{3}j_{3}j_{4}j_{5}j_{6}}, \end{array}$$ (17e)

$$\begin{array}{cc} \lambda_{6} & =\frac{R_{0}-1}{\lambda_{4}\lambda_{5}j_{3}}, \end{array}$$ (17f)

$$\begin{array}{cc} \lambda_{7} & =-\mu , \end{array}$$ (17g)

where,

$$\begin{array}{c} M=S^{0}\beta_{3}\beta_{7}j_{5}+S^{0}\beta_{3}\beta_{6}\beta_{9}. \end{array}$$

Considering R_0_ < 1, all the eigenvalues λ_*i*_ are negative for *i* = 1, 2,…,7. Consequently, when R_0_ < 1 the system of equations of the model (2) is locally asymptotically stable at the disease free equilibrium (DFE) point *P*_0_.

### 5.2 Global stability at DFE

To demonstrate that the model (2) at DFE state is globally stable, the Castillo-Chavez [41] approach is applied [30, 34, 40]. Using the technique introduced by Castillo-Chavez et al., we reproduce our model in the form of following equations.

$$\begin{array}{c} \begin{array}{cc} \frac{dY}{dt} & =K(Y,Z),\\ \frac{dZ}{dt} & =G(Y,Z),\;G(Y,0)=0. \end{array} \end{array}$$ (18)

Where the number of persons who are not affected is indicated by Y=(S,V) and Z=(E,I,C,H) indicates the number of individuals having infection. The last equation of the model is ignored since other equations do not dependent on it. Here, $P_{0}=\left(Y_{0},0\right)$ is the disease free equilibrium point.

To verify the GAS of DFE point by the Castillo-Chavez technique, the below mentioned requirements (C1) and (C2) must be fulfilled.

$$\begin{array}{c} \left(C1\right)\;\text{For}\;\frac{dY}{dt}=K\left(Y_{0},0\right)=0,\;Y_{0}\;\text{is}\;GAS, \end{array}$$ (19)

$$\begin{array}{c} \left(C2)\;G(Y,Z\right)=BZ-\bar{M}\left(Y,Z\right),\;\text{where}\;\bar{M}\left(Y,Z\right)\geq 0\;\text{for}\;\text{all}\;\left(Y,Z\right)\in \Omega , \end{array}$$ (20)

where $B=D_{Z}G\left(Y_{0},0\right)$ is an M-matrix, and Ω represents the model’s feasible region. Thus according to Castillo-Chavez et. al., when the system of Eq (18) satisfies the above mentioned conditions (C1) and (C2), the following theorem holds valid.

**Theorem 10** *The GAS is disease free equilibrium point* *P*_0_ *of the proposed model, if* R_0_ < 1 *and the conditions (C1) and (C2) are satisfied*.

**Proof:** Suppose Y=(S,V) represents uninfected individuals, while Z=(E,I,C,H) symbolize for those who are exposed *E*(*t*), acute infected *I*(*t*), carrier infected *C*(*t*) and hospitalized *H*(*t*), and $P_{0}=\left(Z_{0},0\right)$ is the DFE. Then

$$\begin{array}{c} \frac{dY}{dt}=K\left(Y,Z\right)=[\begin{array}{c} \Pi -\left(\beta_{1}I+\left(\beta_{2}+\nu \right)C+\beta_{3}H\right)S-\beta_{4}S-\mu S\\ \beta_{4}S-\phi_{1}VI-\left(\phi_{2}+\mu \right)V \end{array}]. \end{array}$$ (21)

At $P_{0}=\left(Y_{0},0\right)$, we get

$$\begin{array}{c} K\left(Y_{0},0\right)=[\begin{array}{c} \Pi -\beta_{4}S^{0}-\mu S^{0}\\ \beta_{4}S^{0}-\left(\phi_{2}+\mu \right)V^{0} \end{array}]=0. \end{array}$$ (22)

Since, $Y_{0}=\left(S^{0},V^{0}\right)=(\frac{\Pi }{\mu +\beta_{4}},\frac{\beta_{4}\Pi }{\left(\phi_{2}+\mu \right)\left(\mu +\beta_{4}\right)})$. Thus, $Y_{0}$ is GAS.

Now,

$$\begin{array}{cc} \frac{dZ}{dt} & =BZ-\bar{M}\left(Y,Z\right)\\ = & \left[\begin{array}{cccc} -(\beta_{5}+\mu ) & \beta_{1}S_{0}+\phi_{1}V_{0} & \left(\beta_{2}+\nu \right)S_{0} & \beta_{3}S_{0}\\ \beta_{5} & -(\beta_{6}+\beta_{7}+\beta_{8}\delta_{I}+\mu ) & 0 & 0\\ 0 & \beta_{6} & -(\beta_{9}+\beta_{10}+\mu +\delta_{C}) & 0\\ 0 & \beta_{7} & \beta_{9} & -(\beta_{11}+\mu +\delta_{H}) \end{array}][\begin{array}{c} E\\ I\\ C\\ H \end{array}\right]\\ & -[\begin{array}{c} \left(\beta_{1}+\beta_{2}+\beta_{3}+\nu \right)\left(S_{0}-S\right)\\ \phi_{1}\left(V_{0}-V\right)\\ 0\\ 0 \end{array}], \end{array}$$ (23)

where

$$B=[\begin{array}{cccc} -(\beta_{5}+\mu ) & \beta_{1}S_{0}+\phi_{1}V_{0} & \left(\beta_{2}+\nu \right)S_{0} & \beta_{3}S_{0}\\ \beta_{5} & -(\beta_{6}+\beta_{7}+\beta_{8}\delta_{I}+\mu ) & 0 & 0\\ 0 & \beta_{6} & -(\beta_{9}+\beta_{10}+\mu +\delta_{C}) & 0\\ 0 & \beta_{7} & \beta_{9} & -(\beta_{11}+\mu +\delta_{H}) \end{array}],\;Z=[\begin{array}{c} E\\ I\\ C\\ H \end{array}],$$

and

$$\bar{M}\left(Y,Z\right)=[\begin{array}{c} \left(\beta_{1}+\beta_{2}+\beta_{3}+\nu \right)\left(S_{0}-S\right)\\ \phi_{1}\left(V_{0}-V\right)\\ 0\\ 0 \end{array}].$$

Matrix B indicate that it is a M-matrix. As at disease free equilibrium point *S* ≤ *S*_0_ and *V* ≤ *V*_0_ therefore $\bar{M}\left(Y,Z\right)\geq 0$. Consequently, disease free equilibrium point *P*_0_ is globally asymptotically stable.

### 5.3 Global stability at EE

To prove Global stability at endemic equilibrium (EE) point, we used Lyapunov technique as given in [31, 32]

**Theorem 11** *The EE point represented by* *P*_1_ *is stable if* R_0_ > 1 and it remains unstable if R_0_ < 1.

**Proof:** Suppose the reproductive number R_0_ > 1 so that the EE point exists. Now, we construct a following Volterra type Lyapunov functional *ϕ*, i.e.,

$$\begin{array}{cc} \phi (S,V,E,I,C,H,R)= & \left(S-S^{1}-S^{1}log\;(\frac{S}{S^{1}}))+(V-V^{1}-V^{1}log\;(\frac{V}{V^{1}})\right)\\ & +(E-E^{1}-E^{1}log\;(\frac{E}{E}))+(I-I^{1}-I^{1}log\;(\frac{I}{I^{1}}))\\ & +(C-C^{1}-C^{1}log\;(\frac{C}{C^{1}}))+(H-H^{1}-H^{1}\;log\;(\frac{H}{H^{1}}))\\ & +(R-R^{1}-R^{1}log\;(\frac{R}{R^{1}})). \end{array}$$ (24)

Taking the derivative of *ϕ* with respect to time, we get

$$\begin{array}{cc} \frac{d\phi }{dt}= & (\frac{S-S^{1}}{S})\frac{dS}{dt}+(\frac{V-V^{1}}{V})\frac{dV}{dt}+(\frac{E-E^{1}}{E})\frac{dE}{dt}+(\frac{I-I^{1}}{I})\frac{dI}{dt}\\ & +(\frac{C-C^{1}}{A})\frac{dC}{dt}+(\frac{H-H^{1}}{H})\frac{dH}{dt}+(\frac{\left(R-R^{1}\right)}{R})\frac{dR}{dt}. \end{array}$$

By using the equations of the state model (2), we obtain

$$\begin{array}{cc} \frac{d\phi }{dt}= & \left(\frac{S-S^{1}}{S})(\Pi -cS-\beta_{4}S-\mu S)+(\frac{V-V^{1}}{V})(\beta_{4}S-\phi_{1}VI-\left(\phi_{2}+\mu \right)V\right)\\ & +(\frac{E-E^{1}}{E})(\left(\beta_{1}I+\left(\beta_{2}+\nu \right)C+\beta_{3}H\right)S+\phi_{1}VI-\left(\beta_{5}+\mu \right)E)\\ & +(\frac{I-I^{1}}{I})(\beta_{5}E-\left\{\left(\beta_{6}+\beta_{7}+\beta_{8}+\mu +\delta_{I}\right)\right\}I)\\ & +(\frac{C-C^{1}}{C})(\beta_{6}I-\left\{\left(\beta_{9}+\beta_{10}+\mu +\delta_{C}\right)\right\}C)\\ & +(\frac{H-H^{1}}{H})(\beta_{7}I+\beta_{9}C-\left\{\left(\beta_{11}+\delta_{H}+\mu \right)\right\}H)\\ & +(\frac{R-R^{1}}{R})(\beta_{8}I+\beta_{10}C+\beta_{11}H+\phi_{2}V-\mu R), \end{array}$$

where

$$c=(\beta_{1}I+\left(\beta_{2}+\nu \right)C+\beta_{3}H).$$

After rearranging the terms, we have the following form.

$$\begin{array}{cc} \frac{d\phi }{dt}= & [\Pi +(c+\beta_{4}+\mu )\frac{{S^{1}}^{2}}{S}+q\wedge +(\phi_{1}+\phi_{2}+\mu )\frac{{V^{1}}^{2}}{V}+cS+\phi_{1}VI+(\beta_{5}+\mu )\frac{{E^{1}}^{2}}{E}\\ & +\beta_{5}E+(\beta_{6}+\beta_{7}+\beta_{8}+\mu +\delta_{I})\frac{{I^{1}}^{2}}{I}+\beta_{6}I+(\beta_{9}+\beta_{10}+\mu +\delta_{C})\frac{{C^{1}}^{2}}{C}+\beta_{7}I+\beta_{9}C\\ & +(\beta_{11}+\delta_{H}+\mu )\frac{{H^{1}}^{2}}{H}+\beta_{8}I+\beta_{10}C+\beta_{11}H+\phi_{2}V+\mu \frac{{R^{1}}^{2}}{R}]\\ & -[(c+\beta_{4}+\mu )\frac{{\left(S-S^{1}\right)}^{2}}{S}+(c+\beta_{4}+\mu )S^{1}+\phi \frac{S^{1}}{S}+(\phi_{1}+\phi_{2}+\mu )\frac{{\left(V-V^{1}\right)}^{2}}{V}\\ & +(\phi_{1}+\phi_{2}+\mu )V^{1}+\beta_{4}S\frac{V^{1}}{V}+(\beta_{5}+\mu )\frac{{\left(E-E^{1}\right)}^{2}}{E}+(cS+\phi_{1}VI)\frac{E^{1}}{E}\\ & +\frac{{\left(I-I^{1}\right)}^{2}}{I}(\beta_{6}+\beta_{7}+\beta_{8}+\mu +\delta_{I})+\beta_{5}E\frac{I^{1}}{I}+(\beta_{6}+\beta_{7}+\beta_{8}+\mu +\delta_{I})I^{1}\\ & +\frac{{\left(C-C^{1}\right)}^{2}}{I}(\beta_{9}+\beta_{10}+\mu +\delta_{C})+\beta_{6}I\frac{C^{1}}{C}-(\beta_{9}+\beta_{10}+\mu +\delta_{C})C^{1}+(\beta_{5}+\mu )E^{1}\\ & +\frac{{\left(H-H^{1}\right)}^{2}}{H}(\beta_{11}+\delta_{H}+\mu )+(\beta_{4}I+\beta_{9}C)\frac{H^{1}}{H}+(\beta_{11}+\delta_{H}+\mu )\bar{H}\\ & +\frac{{\left(R-R^{1}\right)}^{2}}{R}\mu +\mu R^{1}+\frac{R^{1}}{R}(\beta_{8}I+\beta_{10}C+\beta_{11}H+\phi_{2}V)]. \end{array}$$

Now it can be written as $\frac{d\phi }{dt}=\theta_{1}-\theta_{2}.$ where

$$\begin{array}{cc} \theta_{1}= & [\Pi +(c+\beta_{4}+\mu )\frac{{S^{1}}^{2}}{S}+q\wedge +(\phi_{1}+\phi_{2}+\mu )\frac{{V^{1}}^{2}}{V}+cS+\phi_{1}VI+(\beta_{5}+\mu )\frac{{E^{1}}^{2}}{E}\\ & +\beta_{5}E+(\beta_{6}+\beta_{7}+\beta_{8}+\mu +\delta_{I})\frac{{I^{1}}^{2}}{I}+\beta_{6}I+(\beta_{9}+\beta_{10}+\mu +\delta_{C})\frac{{C^{1}}^{2}}{C}+\beta_{7}I+\beta_{9}C\\ & +(\beta_{11}+\delta_{H}+\mu )\frac{{H^{1}}^{2}}{H}+\beta_{8}I+\beta_{10}C+\beta_{11}H+\phi_{2}V+\mu \frac{{R^{1}}^{2}}{R}], \end{array}$$

and

$$\begin{array}{cc} \theta_{2}= & [(c+\beta_{4}+\mu )\frac{{\left(S-S^{1}\right)}^{2}}{S}+(c+\beta_{4}+\mu )S^{1}+\phi \frac{S^{1}}{S}+(\phi_{1}+\phi_{2}+\mu )\frac{{\left(V-V^{1}\right)}^{2}}{V}\\ & +(\phi_{1}+\phi_{2}+\mu )V^{1}+\beta_{4}S\frac{V^{1}}{V}+(\beta_{5}+\mu )\frac{{\left(E-E^{1}\right)}^{2}}{E}+(cS+\phi_{1}VI)\frac{E^{1}}{E}\\ & +\frac{{\left(I-I^{1}\right)}^{2}}{I}(\beta_{6}+\beta_{7}+\beta_{8}+\mu +\delta_{I})+\beta_{5}E\frac{I^{1}}{I}+(\beta_{6}+\beta_{7}+\beta_{8}+\mu +\delta_{I})I^{1}\\ & +\frac{{\left(C-C^{1}\right)}^{2}}{I}(\beta_{9}+\beta_{10}+\mu +\delta_{C})+\beta_{6}I\frac{C^{1}}{C}-(\beta_{9}+\beta_{10}+\mu +\delta_{C})C^{1}+(\beta_{5}+\mu )E^{1}\\ & +\frac{{\left(H-H^{1}\right)}^{2}}{H}(\beta_{11}+\delta_{H}+\mu )+(\beta_{4}I+\beta_{9}C)\frac{H^{1}}{H}+(\beta_{11}+\delta_{H}+\mu )\bar{H}\\ & +\frac{{\left(R-R^{1}\right)}^{2}}{R}\mu +\mu R^{1}+\frac{R^{1}}{R}(\beta_{8}I+\beta_{10}C+\beta_{11}H+\phi_{2}V)]. \end{array}$$ (3)

Because all of the parameters are non-negative, we have $\frac{d\phi }{dt}<0$ when *θ*_1_ < *θ*_2_ and $\frac{d\phi }{dt}=0$ if and only if *θ*_1_ = *θ*_2_. The second case suggests that *S* = *S*^1^, *V* = *V*^1^, *E* = *E*^1^, *I* = *I*^1^, *C* = *C*^1^, *H* = *H*^1^, and *R* = *R*^1^.

Therefore, by using LaSalle’s invariance principle [27, 40], the endemic equilibrium point of proposed model is globally asymptotically stable.

## 6. Optimal control problem

In this section, our objective is to create an optimal control problem by formulating a strategy to vaccinate susceptible individuals and to treat or hospitalize those having acute and chronic infectious disease. The model assumes that the rate at which susceptible individuals are vaccinated is time-dependent and denoted by *u*_1_, while the controls for providing treatment to acute and chronic infectious individuals are also time-dependent and are respectively represented by *u*_2_ and *u*_3_. With these considerations, we update the model (2) to obtain a new system of equations with continuous controls. The purpose of these considerations is to give some reliable control strategies for HBV disease control through vaccination and treatment.

### 6.1 Modified HBV model

In this section, we update the proposed model (2) by replacing the three constant parameters *β*_4_, *β*_7_, *β*_9_ as time dependent controls *u*_1_, *u*_2_, *u*_3_. These controls are respectively the vaccination for susceptible and treatments for both acute and chronic HBV patients. In treatment, we care to maintain the comfort and adequate nutritional balance, including replacement of fluids for acute HBV patient and interferon therapy and antiviral medications that are the main treatments for HBV chronic/carriers. Interferon therapy involves injecting interferon into the body to help stimulate the immune system to fight the virus. The drugs like entecavir, tenofovir, and lamivudine can also help to reduce viral load and prevent the liver damage. Hence, the purposed mathematical model (2) is now updated as follows:

$$\begin{array}{cc} \frac{dS}{dt} & =\Pi -\left(\beta_{1}I+\left(\beta_{2}+\nu \right)C+\beta_{3}H\right)S-u_{1}\left(t\right)S-\mu S,\\ \frac{dV}{dt} & =u_{1}\left(t\right)S-\phi_{1}VI-\left(\phi_{2}+\mu \right)V,\\ \frac{dE}{dt} & =(\beta_{1}I+\left(\beta_{2}+\nu \right)C+\beta_{3}H)S+\phi_{1}VI-\left(\beta_{5}+\mu \right)E,\\ \frac{dI}{dt} & =\beta_{5}E-\left(\beta_{6}+u_{2}\left(t\right)+\beta_{8}+\mu +\delta_{I}\right)I,\\ \frac{dC}{dt} & =\beta_{6}I-\left(u_{3}\left(t\right)+\beta_{10}+\mu +\delta_{C}\right)C,\\ \frac{dH}{dt} & =u_{2}\left(t\right)I+u_{3}\left(t\right)C-\left(\beta_{11}+\delta_{H}+\mu \right)H,\\ \frac{dR}{dt} & =\beta_{8}I+\beta_{10}C+\beta_{11}H+\phi_{2}V-\mu R, \end{array}$$ (25)

with the set of non-negative initial conditions:

$$\begin{array}{c} S\left(0\right)=S_{0},\;V\left(0\right)=V_{0},\;E\left(0\right)=E_{0},\;I\left(0\right)=I_{0},\;C\left(0\right)=C_{0},\;H\left(0\right)=H_{0},\;R\left(0\right)=R_{0}. \end{array}$$

Now we construct an optimal control problem with an aim to determine optimal vaccination and treatment rates to control the disease. For this, we define an objective functional that involves the infected state variables and control variables with an objective to minimize it. In addition, we use Pontryagin’s maximum principle to drive the necessary conditions to evaluate the defined optimal control problem [34, 35, 40].

### 6.2 Objective functional

We define the following objective functional to be minimized:

$$\begin{array}{c} J(Z,u)=\int_{0}^{T_{f}}(A_{1}I\left(t\right)+A_{2}C\left(t\right)+A_{3}H\left(t\right)+\frac{1}{2}w_{1}u_{1}^{2}\left(t\right)+\frac{1}{2}w_{2}u_{2}^{2}\left(t\right)+\frac{1}{2}w_{3}u_{3}^{2}\left(t\right))dt, \end{array}$$ (26)

where *T*_*f*_ represents the final time, *I*(*t*), *C*(*t*), and *H*(*t*) are state and *u*(*t*) = (*u*_1_, *u*_2_, *u*_3_) = (*β*_4_, *β*_7_, *β*_9_) is time-dependent control. The first one of the control variable *u*_1_(*t*) is vaccination rate of susceptible, the second *u*_2_(*t*) and third *u*_3_(*t*) represent treatment rate of acute infectious *I*(*t*) and carrier infectious *C*(*t*) respectively. Non-negative constants *A*_*i*_, *i* = 1, 2, 3 are weights associated with state variables and *w*_*j*_, *j* = 1, 2, 3 are costs of controls.

Our purpose is to determine the optimal controls $u_{1}^{*},u_{2}^{*},u_{3}^{*}$ ∈ *U* in such a way that the objective functional (26) get minimized, i.e.,

$$\begin{array}{c} \underset{u\in U}{\text{min}}\;J\left(Z,u\right)\;\text{subject}\;\text{to}\;\text{the}\;\text{system}\;\text{of}\;\text{Eq.}\;\left(25\right). \end{array}$$ (27)

Here *U* denotes the control set, which is specified as

$$\begin{array}{c} U=\{u\left(t\right)|0\leq u_{i}\left(t\right)\leq 1,i=1,2,3\;\wedge \;0\leq t\leq T_{f}\}. \end{array}$$

### 6.3 Necessary conditions

We implement Pontryagin’s maximum principle [35] to develop the conditions that are necessary for optimization of the given control problem (27). These conditions are deduced from Hamiltonian H. It is stated as the sum of integrand of objective functional and the right hand sides of each of the equations of the proposed model (25) multiplied by adjoint variables, i.e.,

$$\begin{array}{c} H\left(t,Z,u,\psi \right)=\sigma \left(Z,u\right)+\sum_{j=1}^{7}\psi_{j}g_{j}\left(t,Z,u\right), \end{array}$$

where $\sigma =A_{1}I\left(t\right)+A_{2}C\left(t\right)+A_{3}H\left(t\right)+\frac{1}{2}w_{1}u_{1}^{2}\left(t\right)+\frac{1}{2}w_{2}u_{2}^{2}\left(t\right)+\frac{1}{2}w_{3}u_{3}^{2}\left(t\right)$,

*Z* = (*S*, *V*, *E*, *I*, *C*, *H*, *R*) denotes the state variables, *ψ*_*j*_ *j* = 1, 2, 3,…,7 are the corresponding adjoint variables and *g_j_* (*t*, *Z*, *u*), *j* = 1, 2, 3,…,7 represent the right hands side of system (25).

Thus, the Hamiltonian function for the optimal control problem (27) can be expressed as:

$$\begin{array}{cc} H(t,Z,u,\psi )= & A_{1}I\left(t\right)+A_{2}C\left(t\right)+A_{3}H\left(t\right)+\frac{1}{2}w_{1}u_{1}^{2}\left(t\right)+\frac{1}{2}w_{2}u_{2}^{2}\left(t\right)+\frac{1}{2}w_{3}u_{3}^{2}\left(t\right)\\ & +\psi_{1}(\Pi -(\beta_{1}I+\left(\beta_{2}+\nu \right)C+\beta_{3}H)S-\left(\mu +\beta_{4}\right)S)\\ & +\psi_{2}(\beta_{4}S-\phi_{1}VI-\left(\phi_{2}+\mu \right)V)\\ & +\psi_{3}(\left(\beta_{1}I+\left(\beta_{2}+\nu \right)C+\beta_{3}H\right)S+\phi_{1}VI-\left(\beta_{5}+\mu \right)E)\\ & +\psi_{4}(\beta_{5}E-\left(\beta_{6}+\beta_{7}+\beta_{8}+\beta_{9}+\delta_{I}+\mu \right)I)\\ & +\psi_{5}(\beta_{6}I-\left(\beta_{9}+\beta_{10}+\delta_{C}+\mu \right)C)\\ & +\psi_{6}(\beta_{7}I+\beta_{9}C-\left(\beta_{11}+\delta_{H}+\mu \right)H)\\ & +\psi_{7}(\beta_{8}I+\beta_{10}C+\beta_{11}H+\phi_{2}V-\mu R). \end{array}$$ (28)

The first optimality condition:

$$\begin{array}{c} \frac{\partial H}{\partial u}=0, \end{array}$$

of the Pontryagin maximum principle offers us the below equations for control variables:

$$\begin{array}{c} u_{1}=\frac{S(\psi_{1}-\psi_{2})}{w_{1}},\\ u_{2}=\frac{I(\psi_{4}-\psi_{6})}{w_{2}},\\ u_{3}=\frac{C(\psi_{5}-\psi_{6})}{w_{3}}, \end{array}$$

and the next expressions are updated controls with bounded restrictions, i.e.,

$$\begin{array}{c} u_{1}=min\{1,max\{0,\frac{S(\psi_{1}-\psi_{2})}{w_{1}}\}\},\\ u_{2}=min\{1,max\{0,\frac{I(\psi_{4}-\psi_{6})}{w_{2}}\}\},\\ u_{3}=min\{1,max\{0,\frac{C(\psi_{5}-\psi_{6})}{w_{3}}\}\}. \end{array}$$ (29)

The optimality second condition

$$\begin{array}{c} \frac{\partial H}{\partial Z_{j}}=-\frac{d\psi_{j}}{dt},\;j=1,2,3,\ldots ,7, \end{array}$$

of the Pontryagin maximum principle yields the following system of linear adjoint equations:

$$\begin{array}{cc} \frac{d\psi_{1}}{dt}= & (\beta_{1}I+\left(\beta_{2}+\nu \right)C+\beta_{3}H)\psi_{1}\\ & -(\beta_{1}I+\left(\beta_{2}+\nu \right)C+\beta_{3}H)\psi_{3}+\beta_{4}\left(\psi_{1}-\psi_{2}\right)+\mu \psi_{1},\\ \frac{d\psi_{2}}{dt}= & (\phi_{1}I)\psi_{2}-(\phi_{1}I)\psi_{3}+\phi_{2}\left(\psi_{2}-\psi_{7}\right)+\mu \psi_{2},\\ \frac{d\psi_{3}}{dt}= & \beta_{5}\left(\psi_{3}-\psi_{4}\right)+\mu \psi_{3},\\ \frac{d\psi_{4}}{dt}= & \beta_{1}S\left(\psi_{1}-\psi_{3}\right)+\phi_{1}V\left(\psi_{2}-\psi_{3}\right)+\beta_{6}\left(\psi_{4}-\psi_{5}\right)+\beta_{7}\left(\psi_{4}-\psi_{6}\right)\\ & +\beta_{8}\left(\psi_{4}-\psi_{7}\right)+\left(\mu +\delta_{I}\right)\psi_{4}-A_{1},\\ \frac{d\psi_{5}}{dt}= & S\left(\beta_{2}+\nu \right)\left(\psi_{1}-\psi_{3}\right)+\beta_{9}\left(\psi_{5}-\psi_{6}\right)+\beta_{10}\left(\psi_{5}-\psi_{7}\right)\\ & +\left(\mu +\delta_{C}\right)\psi_{5}-A_{2},\\ \frac{d\psi_{6}}{dt}= & S\beta_{3}\left(\psi_{1}-\psi_{3}\right)+\beta_{11}\left(\psi_{6}-\psi_{7}\right)+\left(\delta_{H}+\mu \right)\psi_{6}-A_{3},\\ \frac{d\psi_{7}}{dt}= & \mu \psi_{7}, \end{array}$$ (30)

along with final conditions:

$$\begin{array}{c} \psi_{j}\left(T_{f}\right)=0,\;j=1,2,3,\ldots ,7. \end{array}$$ (31)

The derivative of the given Hamiltonian H with respect to the adjoint variables *ψ_j_*, *j* = 1, 2, 3,…,7, lead us to system of state Eq (25). We implement the following algorithm through MATLAB code to find an optimal solution of the control problem (27).

### 6.4 Solution algorithm

We implement the following algorithm through MATLAB code to find an optimal solution of the control problem (27).

**Algorithm 1**

1. *Begin with* *m* = 0 *and take a supposed value for control variable* *u*_*m*_ ∈ *U*.

2. *Resolve the system of* Eqs (25) *and the associated adjoint system* (30) *by using the control* *u*_*m*_.

3. *Calculate* *u*_*new*_ *by using the definition of bounded optimal controls* (29).

4. *Use the relation* *u*_*m*_ = (*u*_*new*_ + *u*_*m*_)/2, *to update the control* *u*_*m*_.

5. *If* $\frac{\|\phi_{m}-\phi_{m-1}\|}{\|\phi_{m}\|}<\text{tol}.$ *for* *m* > 0, *then STOP here, otherwise* *m* → *m* + 1 *and go to step 2*.

The state variables *Z_j_*, *j* = 1, 2,…,7, adjoint variables *ψ_j_*, *j* = 1, 2,…,7, and control variables *u*_*i*_, *i* = 1, 2, 3 are all represented by *ϕ*. In step 5, tolerance *tol*. is established for convergence of the algorithm.

### 6.5 Optimal solutions

The solutions are determined by using the above mentioned Algorithm 1 as well as a MATLAB code. The state *Z*_*j*_ and adjoint *ψ_j_*, *j* = 1, 2,…,7 variables are calculated by implementing the Runge-Kutta method of order 4. Simpson’s $\frac{1}{3}$ rule is used to approximate the objective functional (26). Here we consider three optimal control strategies to minimize the HBV disease in the population. These strategies are explained below with the help of figures.

### Case 1: Control with vaccine

**Fig 3:**
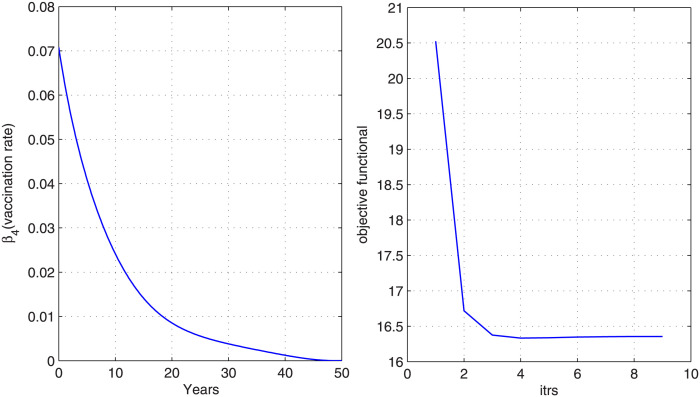
Optimal control variable and corresponding cost functional. The cost functional has reached to its minimum with the optimal vaccine rate *β*_4_.

**Fig 4:**
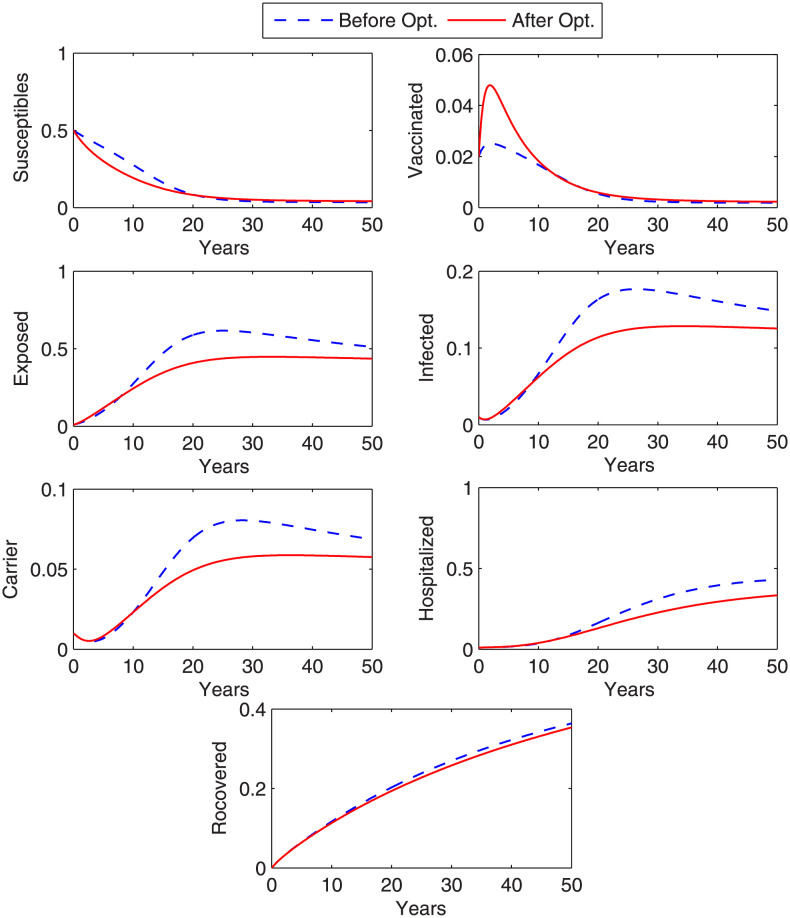
Optimized state variables. Figure shows state variables before and after optimization. A decrease in the infected and carrier individuals is noticed with the implemented vaccine strategy.

In the first strategy, we control the disease at the population level with just one control that is vaccine. Fig 3 shows the graph of optimal control along with associated objective functional. We observe from the graph that objective functional attains its minimum value in nine iterations. Under this optimal control (optimal vaccine rate *β*_4_), the state variables before and after optimization are plotted and shown in Fig 4. We notice from Fig 4 that the size of exposed, infected, chronic carriers and hospitalized class has a significant decrease after optimization with optimal vaccination rate *β*_4_. For this strategy, we need high vaccination for the first ten years.

### Case 2: Control with treatment

**Fig 5:**
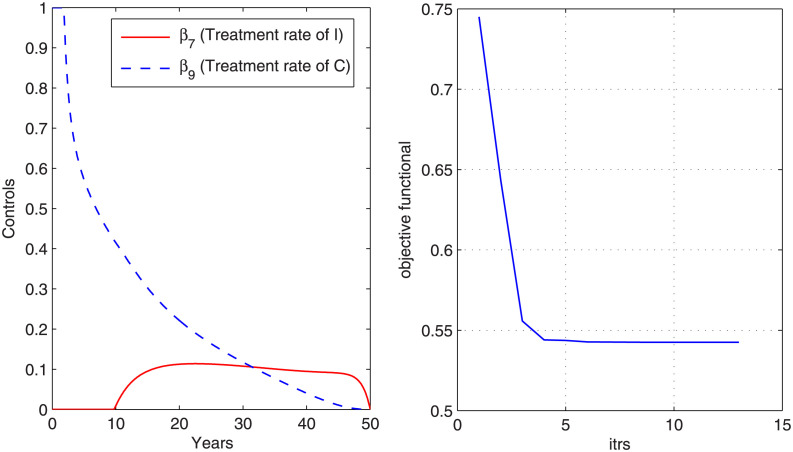
Optimal control variables and corresponding cost functional. The figure shows optimal controllers that have minimized the cost functional with treatment strategy.

**Fig 6:**
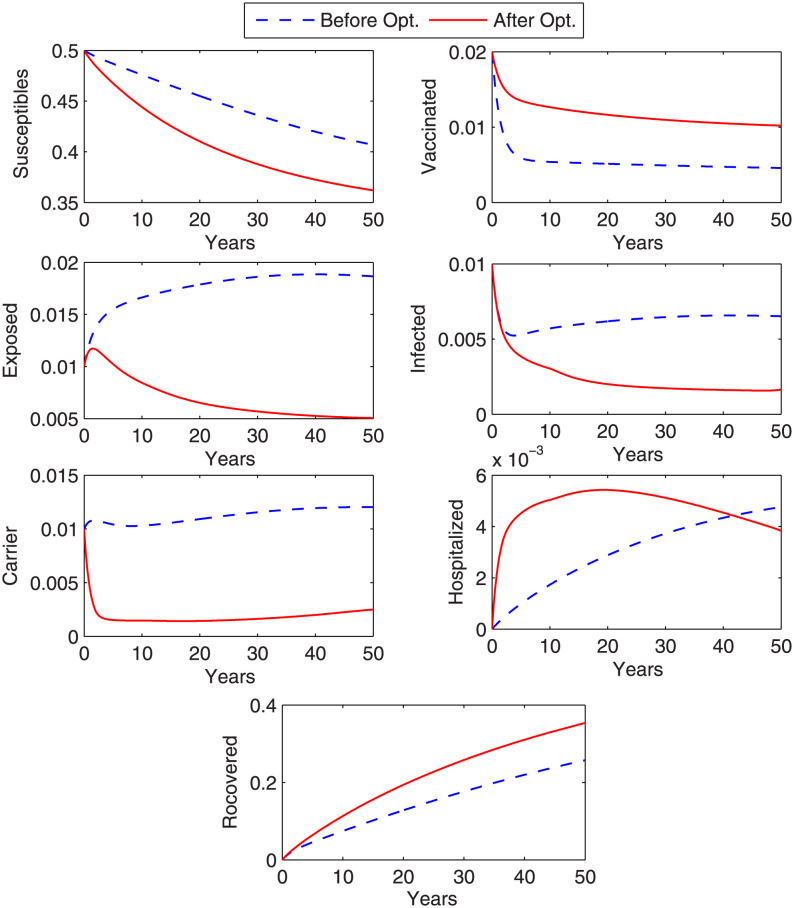
Optimized state variables. Figure shows state variables before and after optimization. A remarkable decrease in the exposed, infected and carrier individuals is noticed with the implemented treatment strategy.

In this case, the control that is considered to overcome the disease is treatment. Fig 5 shows the graph of objective functional along with the graph of corresponding optimal controls *β*_7_ (treatment rate of I) and *β*_9_ (treatment rate of C). We observe from the graph that objective functional attains its minimum value in thirteen iterations under the influence of optimal controls that vary with time. It is noticed that more treatment of chronic carriers is required as compared to the treatment for infected (acute) individuals. The graphs in Fig 6 shows the state variables before and after optimization. From Fig 6, we can conclude that the number of exposed, infected, chronic carrier individuals decrease after optimization under optimal control variable. However, the hospitalized individuals increase in the beginning and then decrease day by day. The results obtained for case 2 in controlling disease are better than the result of case 1.

### Case 3: Control with vaccine and treatment

**Fig 7:**
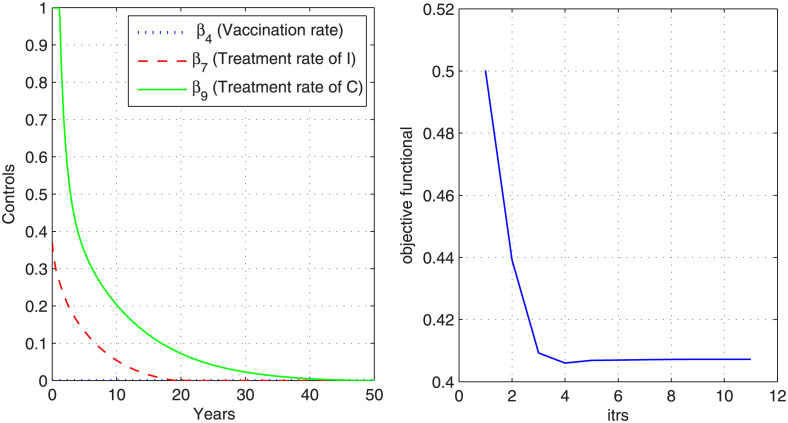
Optimal control variables and corresponding cost functional. Figure shows the optimal vaccine and treatment rates that have minimized the cost functional.

**Fig 8:**
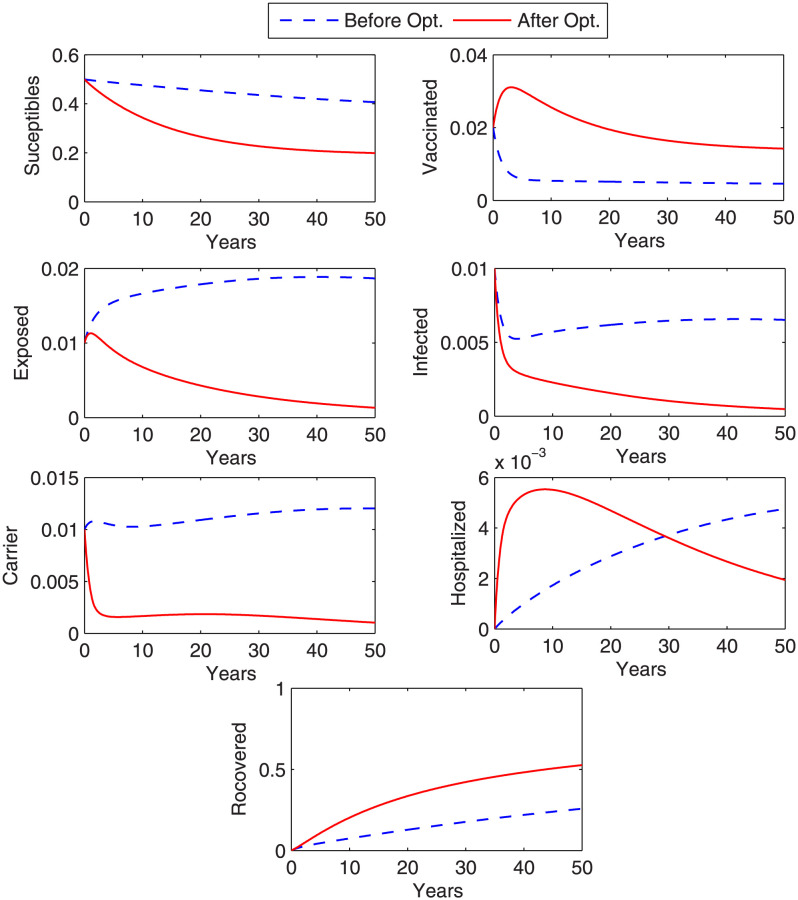
Optimized state variables. Figure shows optimal behaviour of state variables before and after optimization. A remarkable decrease in the exposed, infected and carrier individuals is noticed under the strategy when both vaccination rate and treatment rates are considered together as control variables.

In this case, we control the disease by considering all controls together, i.e., vaccine and treatment rates. Fig 7 describes the graph objective functional. From graph, we observe that the objective functional attains its minimum value in eleven iterations under the effect of optimal control variables. The optimal controls *β*_4_ (vaccination rate), *β*_7_ (treatment rate of I) and *β*_9_ (treatment rate of C) that minimize the objective functional are also plotted in Fig 7. The graphs in Fig 8 shows the state variables with and without optimization. From Fig 8, we observe that there is a remarkable decrease in the curves of exposed, infected, chronic carriers and hospitalized after optimizations under optimal controls (vaccine and treatment rates).

The analysis reveals that the exposed, infected and chronic carriers can be reduced by implementing these control strategies. However, the graph shows that the treatment strategy is more effective in reducing the exposed, infected and chronic carriers as compared to the vaccination strategy.

## 7. Conclusions

In this current analysis, we discussed a newly designed HBV disease model SVEICHR along with the optimal control strategies for disease control. We proved that the proposed model has unique solutions which are positive and bounded. We determined the model’s disease free point and endemic equilibrium points. To analyze the dynamics of epidemic in the population, we computed the reproduction number R_0_. Then, at disease free equilibrium point, we also checked the local and global stability of the model. We also examined the global stability of system of equations at endemic equilibrium point and found that our system is stable there.

For possible eradication of disease in the population, we defined an objective functional to be minimized. We used the Pontryagin’s maximum principle to develop the optimality conditions. Then to find the optimal solutions of the given control problem, the MATLAB software is used for coding. We discussed three strategies to control the HBV disease optimally. As a first strategy, we analyzed the effect on control of disease by vaccination. In the second case, we studied the optimal control problem under the influence of only treatment. Thirdly, we put all the strategies (vaccine and treatment) together and visualized their significance on control of disease. In all these cases, we noticed a significant decrease in the curves of the infected classes after optimization, particularly the third case has significant effect in preventing the spread of disease. We also observed that treatment strategy has more impact on disease control as compared to vaccination strategy.

Our upcoming work will involve demonstrating a clear representation of HBV disease using a fractional model with an ABC derivative operator and a range of intervention strategies. We will also determine the most effective measures for vaccination and hospitalization through analysis of a fractional order optimal control problem.

## Funding

This work was supported by the Deanship of Scientific Research, Vice Presidency for Graduate Studies and Scientific Research, King Faisal University, Saudi Arabia [Grant No. GRANT3716].
